# Population and Demographic Structure of *Ixodes scapularis* Say in the Eastern United States

**DOI:** 10.1371/journal.pone.0101389

**Published:** 2014-07-15

**Authors:** Joyce M. Sakamoto, Jerome Goddard, Jason L. Rasgon

**Affiliations:** 1 Department of Entomology, the Center for Infectious Disease Dynamics, and the Huck Institutes of The Life Sciences, The Pennsylvania State University, University Park, Pennsylvania, United States of America; 2 Department of Biochemistry, Molecular Biology, Entomology and Plant Pathology, Mississippi State University, Mississippi State, Mississippi, United States of America; University of North Dakota School of Medicine and Health Sciences, United States of America

## Abstract

**Introduction:**

The most significant vector of tick-borne pathogens in the United States is *Ixodes scapularis* Say (the blacklegged tick). Previous studies have identified significant genetic, behavioral and morphological differences between northern vs. southern populations of this tick. Because tick-borne pathogens are dependent on their vectors for transmission, a baseline understanding of the vector population structure is crucial to determining the risks and epidemiology of pathogen transmission.

**Methods:**

We investigated population genetic variation of *I. scapularis* populations in the eastern United States using a multilocus approach. We sequenced and analyzed the mitochondrial COI and 16S genes and three nuclear genes (serpin2, ixoderin B and lysozyme) from wild specimens.

**Results:**

We identified a deep divergence (3–7%) in *I. scapularis* COI gene sequences from some southern specimens, suggesting we had sampled a different *Ixodes* species. Analysis of mitochondrial 16S rRNA sequences did not support this hypothesis and indicated that all specimens were *I. scapularis*. Phylogenetic analysis and analysis of molecular variance (AMOVA) supported significant differences between northern vs. southern populations. Demographic analysis suggested that northern populations had experienced a bottleneck/expansion event sometime in the past, possibly associated with Pleistocene glaciation events.

**Conclusions:**

Similar to other studies, our data support the division of northern vs. southern *I. scapularis* genetic lineages, likely due to differences in the demographic histories between these geographic regions. The deep divergence identified in some COI gene sequences highlights a potential hazard of relying solely on COI for species identification (“barcoding”) and population genetics in this important vector arthropod.

## Introduction

In the United States, the most significant vector of tick-borne pathogens is *Ixodes scapularis* Say (the blacklegged tick) [1]. *I. scapularis* transmits multiple zoonotic pathogens including *Borrelia burgdorferi sensu stricto* (Lyme disease), *B. miyamotoi* (tick relapsing fever), *Anaplasma phagocytophilum* (human granulocytic anaplasmosis), *Babesia microti* (babesiosis), and Deer Tick Virus (*I. scapularis* variant of Powassan virus)[1] [2] [3] [4]. Because tick-borne pathogens are dependent on their vectors for transmission, a baseline understanding of the vector population structure is crucial to determining the risks and epidemiology of pathogen transmission.

Multiple DNA sequences have been used to examine the evolution and population genetics of *I. scapularis* [5] [6] [7] [8] [9] [10] [11] [12]. With the sequencing of the *I. scapularis* genome, it has become much easier to screen for novel genetic markers for this species [12]. The taxonomic history of *I. scapularis* has been somewhat contentious, with some researchers claiming that it is actually a species complex consisting of *I. scapularis* in the Southern USA and *I*. *dammini* in the north [13] [14] [15] [16] [17] [7] [8] [18] [19] [20], While scientific consensus has for the most part rejected this interpretation, it is clear that there are significant genetic and demographic differences between northern and southern populations of this tick [21] [3] [12]. To address this issue, we investigated genetic variation in *I. scapularis* populations in the eastern United States using a multilocus approach in which we sequenced and analyzed the mitochondrial COI and 16S genes, and three nuclear genes (serpin 2, ixoderin B and lysozyme).

## Materials and Methods

### Field tick collections

**Figure 1:**
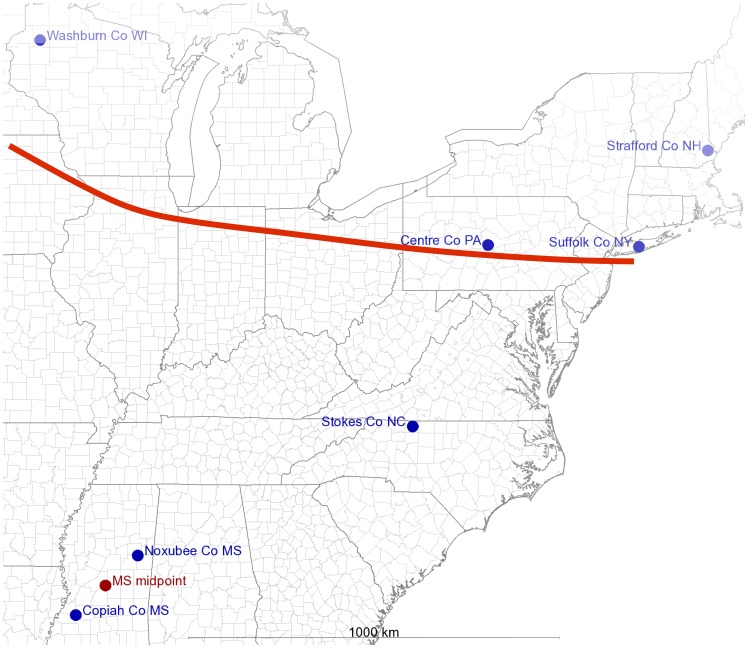
*I. scapularis* collection sites. GPS coordinates of collection sites were plotted onto a public domain map of the United States (http://nationalmap.gov/) using an online GPS generation program (http://www.gpsvisualizer.com). Blue dots denote the collection sites, while the red dot denotes the approximate midpoint between two Mississippi collection sites used for the Mantel test. Red solid line represents approximate extent of historical Pleistocene glacial coverage.

Tick samples were collected during 2006–2012 by flagging with a 1 m^2^ canvas cloth. Samples were catalogued, surface-disinfested, and extracted immediately or stored at −80°C until extraction (see below). *I. scapularis* adults or nymphs were collected from populations from Wisconsin, New Hampshire, Pennsylvania, Mississippi, North Carolina and New York (Table 1, Figure 1). GPS coordinates were inputted into an online GPS generation program to generate a map of the collection sites on a map of the United States (http://www.gpsvisualizer.com; map image from the public domain [http://nationalmap.gov]). No ethical clearance was required to conduct research on invertebrate ectoparasites. All samples were either submitted by private collectors or collected by the authors after obtaining appropriate permissions.

**Table 1:** Tick collection information.

Population sampled by state	Collector	#Samples/State
New Hampshire (Strafford County)	A. Eaton	20 (10 female, 10 male)
Wisconsin (Washborn County)	G. Ebel	20 (10 female, 10 male)
Mississippi (Noxubee and Copiah Counties)*	J. Goddard	20 (14 female, 3 males from Copiah County; 3 F from Noxubee County
Pennsylvania (Centre County)	J. Sakamoto	20 (10 female, 10 male)
New York (Suffolk County)	I. Rochlin	8 (1 female, 1 male, 6 nymphs)
North Carolina (Stokes County)	C. Apperson, B. Harrison	10 males
Wikel colony**	D. Sonenshine	3 (egg masses from 3 individual females)
MSU-A***	J. Goddard	*Ixodes brunneus*
MSU-B***	J. Goddard	*Ixodes scapularis*
MSU-C***	J. Goddard	*Ixodes affinis*
MSU-D***	J. Goddard	*Ixodes scapularis*

*An approximate midpoint GPS was used for population analysis.

**Females were descendants of the Wikel colony used for the Ixodes scapularis genome sequence.

***Species were determined morphologically by JG and independently confirmed by mitochondrial 16S cloning and sequencing by JMS.

### DNA extraction

Prior to DNA extraction, each tick sample was individually surface-disinfested with 95% ethanol for 15 s, 10% bleach for 60 s, washed sequentially in 3 baths of sterile nuclease-free water, and dried on autoclave-sterilized filter paper in a sterile petri plate. Each adult tick was bisected and one half archived at −80°C, while the other half was used for DNA extraction. Nymphs were extracted in their entirety rather than bisected. Bisected samples were frozen briefly (30 min/−80°C), macerated with a sterile micropestle, and the DNA extracted according to the respective manufacturer's guideline. Genomic DNA was extracted from legs or small fragments stored in 95% ethanol from the “unknown” samples (MSU A–D). The ethanol was completely evaporated from the samples prior to DNA extraction. Genomic DNA was extracted from samples using either the DNeasy Blood and Tissue kit (Qiagen 69506) or the GenElute Bacterial Genomic DNA kit (Sigma NA2110), following the manufacturer's guidelines. DNA concentration was determined with a Nanodrop spectrophotometer and adjusted to 5 ng/ul prior to use in PCR. For comparative purposes, genomic DNA was extracted from egg masses from 3 individual females derived from the Wikel colony, which was used to generate the *I. scapularis* whole genome shotgun sequences on Vectorbase.

### Amplification of mitochondrial DNA (cytochrome oxidase c subunit I and 16S)

Primers COI907F and COI907R (Table 2) were designed to amplify a 907 bp fragment of the *I. scapularis* cytochrome c oxidase subunit I gene (GenBank Accn# ABJB010748661.1). Primers were designed using Primer3 [22]. Samples were initially screened using 10 ul reactions contained 1 ul genomic DNA, 0.2 ul each forward and reverse primer (10 mM each), 5.0 ul of 2X Taq Master mix (New England Biolabs, M0270), and 3.6 ul of sterile nuclease-free water. Amplification conditions were as follows: 95°C/5 min, 35 cycles of 95°C/30 s, 60°C/30 s, 72°C/60 s, and a final extension of 72°C/10 minutes. Samples that were positive by PCR were amplified for sequencing in 50 ul reactions using Phusion High-Fidelity DNA Polymerase (New England Biolabs, M0530) as follows: 5 ng of DNA were mixed with 1 ul of dNTP mix (10 mM each), 1 ul each of forward and reverse primers (10 mM), 0.5 ul ( = 0.01 Units) Phusion polymerase, 10 ul HF buffer, and 35 ul sterile nuclease-free water. Samples were amplified as follows: an initial melting cycle at 98°C /30 s, and cycled 35 times at 98°C/30 s, 60°C/30 s, and 72°C/30 s, followed by a final extension of 72°C/10 minutes. PCR products were prepared for sequencing by treating with ExoSAP-IT (USB, Affymetrix Cat# 78201) following manufacturer's guidelines. The samples were sequenced in both directions on an ABI 3130/Genetic Analyzer.

**Table 2:** PCR primer sequences used in this study.

Gene	Primer	5′→3′	Amplicon size	Genbank /Vectorbase	Reference
Cytochrome c oxidase subunit I	COI907F	TTAGGGGCACCAGACATAGC	907	NW_002834719.1 IscW_ISCW013800	This study
	COI907R	TAGCAAAAACGGCTCCTATTG			This study
Mitochondrial 16S rRNA	16S+1	CCGGTCTGAACTCAGATCAAGT	460		Norris et al 1996
	16S-1	CTGCTCAATGATTTTTTAAATTGCTGTGG			Norris et al 1996
Ixoderin B	IxodBe2-3F	ACACGTATGCCTCAAAGTGG	502	NW_002845951.1 IscW_ISCW013797	This study
	IxodBe2-3R	GCACTATATCCAGCGGGAAG			This study
Lysozyme	Lys_eSNP1F	TGTCTTTGGCTTGGATCGTC	512	NW_002737421.1 IscW_ISCW020680	This study
					
	Lys_eSNP1R	ATTCTTCCACCTGCCCTACG			This study
Serpin 2	Serp2Ae1-6_F	TTACGCTCCCGACGTTATTC	651	NW_002630218.1 IscW_ISCW018607	This study
	Serp2Ae1-6_R	TTCGAGGGATCAAACAGGTC			This study

*Primers designed for this study were designed using Primer3*.

Several samples had COI sequences that were highly divergent (see results). A subset of these samples was sequenced at the 16S rRNA gene for comparison to the previously described haplotypes of *I. scapularis* [3] [9] [8]. We amplified a 460 bp fragment using primers 16S+1 and 16S-1 (Table 2), using a modified step-down protocol to eliminate nonspecific products: 94°C, 4 cycles of 94°C/15 s, 58°C/15 s, 68°C/30 s, 4 cycles of 94°C/15 s, 54°C/15 s, 68°C/30 s, and 27 cycles of 94°C/15 s, 50°C/15 s, 68°C/30 s, with a final extension of 68°C/5 minutes. Three samples each from the two divergent COI clusters were sequenced, along with one “typical” haplotype of *I. scapularis* each from Mississippi and Pennsylvania. To confirm that the samples from the two divergent clades were not actually from different species, samples (leg or body fragment) of known species identity were received and analyzed in a blinded manner for cloning (Strataclone PCR cloning kit, Stratagene #240205) and sequencing. All sequences were aligned and compared to the 16S sequences from Rich et al 1995, Norris et al 1996, and Qiu et al 2002 using phylogenetic and population analyses described below [8] [9] [3].

### Amplification of nuclear genes

*I. scapularis* is known to harbor a maternally-inherited endosymbiotic *Rickettsia* [23] [24]. All samples in this study were assayed for the rickettsial outer membrane protein A (rompA) [25] and all tested positive (data not shown). Since there have been studies suggesting that mitochondrial evolution might be affected by endosymbionts [26] [27], we chose to sequence three additional nuclear genes from 4 of the 6 populations assayed for COI. Primers were designed to amplify fragments of three putative innate immune genes, identified through blastn homology to the *I. scapularis* genome and EST sequence data on NCBI and Vectorbase. The three genes were: 1) a salivary gland-specific lectin (ixoderin B, Vectorbase ID IscW_ISCW013797; GenBank NW_002845951.1); 2) a lysozyme (Vectorbase ID IscW_ISCW020680; GenBank NW_002737421.1); and 3) a serine protease inhibitor (serpin 2 precursor, Vectorbase ID IscW_ISCW011017; GenBank NW_002630218.1). Fragments (between 500 and 600 bp) were amplified and sequenced from the Wisconsin, Mississippi, New Hampshire, and North Carolina specimens, and from 5 Wikel colony females.

### Post-sequencing data analysis

For each sample, the aligned sequences were checked against trace files using MEGA 5.2.1 [28]. The consensus of both strands was generated using jEmboss [29]. The consensus sequences were aligned with MUSCLE 3.5 [30] and trimmed to overlapping regions that were as follows: 755 bp for COI, 360 bp for 16S rRNA, 413 bp for ixoderin B, 551 bp for serpin 2, and 396 bp for lysozyme.

### Confirmation of sequences by blastn and the BOLD Identification system tool

Aligned and trimmed sequences were run through blastn (Blast2.2.28+) against all NCBI nucleotide (nt) databases [31]. Additionally, sequences were run through the BOLD identification system (IDS) identification engine [32]. Sequences that had less than 98.0% blast identity match to *I. scapularis* were submitted to the BOLD IDS to assess how closely the sequences matched archived *I. scapularis* COI sequences. The BOLD IDS runs a global alignment of all queries against a COI-specific Hidden Markov Model (HMM), and then compares the results against the reference library. The IDS engine provides species designations if the query is less than 1% divergent from a reference sequence, and genus designations if there is less than 3% divergence. Each sample is used to generate a phylogenetic tree and identification determined based on the tree output.

When several of the Mississippi sequences did not produce a species level identification (based on the BOLD criteria, species level identification is not provided for samples that are more than 1% divergent from a reference sequence), we compared our sample sequences to *I. scapularis* COI sequences from other studies in the GenBank database (Table S1), including the complete coding sequence from th*e I. scapularis* whole genome shotgun sequencing project from which the original COl primer sequences were designed, sequences from *Ixodes scapularis* in Canada, and a sequence from *I. scapularis* used by Noureddine et al 2011 as an outgroup to *I. ricinus* [33] [34]. These were aligned with our sequences and trimmed to 338 bp (overlapping region for all sequences) for phylogenetic analysis.

To confirm our divergent Mississippi sequences were *I. scapularis* and not a sister species, we also sequenced the 16S gene from a subset of the divergent Mississippi samples and compared them phylogenetically to 16S rRNA sequences found on GenBank (Table S2).

### Phylogenetic analysis

Phylogenetic analysis was conducted using MrBayes version 3.2.1 [35]. MrBayes uses a Markov Chain Monte Carlo (MCMC) integration method to estimate posterior probabilities. The evolutionary model implemented for the nucleic acid sequences was estimated using jModelTest [36]. Best models for each gene were as follows: TPM3 (three parameter model) for mitochondrial genes COI and 16S (gamma = 0.1540 and 1.360, respectively); Kimura (K80) for lysozyme (gamma = 0.0940), ixoderin B (no gamma), and model TrN for serpin 2 (equal base frequencies, p-inversion = 0.8870). The MCMC was run until the average standard deviation of split frequencies was below the stop value of 0.01 (using the stoprule option), sampled every 100 generations, with the default of 25% burn-in specified. A 50% majority rule consensus tree was constructed from the remaining 75% of the trees. MrBayes was also used to generate a phylogenetic tree from the concatenated sequences from the 3 nuclear genes. Since each of the genes had different substitution model conditions suggested by jModelTest, the different model conditions were specified for each gene (partition) in MrBayes. For the COI gene, the matching fragment of the complete mitochondrial genome sequence of *Ixodes ricinus* (GenBank Accn# JN248424) was used as outgroup. *Ixodes pacificus* haplotype SSCP CA_2 (GenBank Accn# AF309009) was used as outgroup for the mitochondrial 16S rRNA gene. No suitable outgroup sequences were available for the nuclear genes and these trees were thus left unrooted.

Figtree version 1.3.1 was used to view trees produced by MrBayes (http://tree.bio.ed.ac.uk/software/figtree/). When the 16S rRNA sequence from the blinded sample “MSU-A” (*I. brunneus*) was determined to be more divergent than the chosen root (*I. pacificus*), the tree was left unrooted. A second Bayesian analysis was run with just the 16S rRNA sequences from *I. scapularis* samples and the outgroup *I. pacificus*.

### Population structure, neutrality, diversity, and genetic distance analyses

For the COI gene-based population genetic analysis, Arlequin 3.0 [37] was used to partition genetic variation between Northern USA (NY, WI, NH, PA) and Southern USA (MI, NC) populations using analysis of molecular variance (AMOVA).

An online tool Intrapop Neutrality Test (http://wwwabi.snv.jussieu.fr/achaz/neutralitytest.html) was used to calculate Tajima's D, and Fu and Li's F* and D* statistics [38] [39] [40]. Haplotype diversity/frequency (for COI), was conducted with the MS Excel plugin GenAlEx v 6.5 [41] [42]. Mantel tests were conducted using GenAlEx v 6.5. Because only three samples came from Noxubee County, we pooled the Mississippi samples and estimated the midpoint between the two collection points (http://www.geomidpoint.com/meet/) for Mantel analysis. Demographic analyses were conducted using DnaSP (http://www.ub.edu/dnasp/).

## Results

In total, we obtained clean bi-directional COI sequences from 89 out of 94 specimens assayed from 6 populations (four northern USA: PA, WI, NY, NH; two southern USA: NC, MS). Samples that had clean sequences in only one strand were excluded from phylogenetic analyses. After sequencing, each sample was confirmed to be *Ixodes scapulari*s by blastn homology to GenBank and against the BOLD IDS (described in the Methods). Several of the Mississippi sequences were not assigned species-level identification on the BOLD IDS (Table 3).

**Table 3:** Genbank blastn and BOLD IDS best matches for Mississippi samples.

		Genbank			BOLD IDS		
Sample	Gene	Genbank Accn match	%identity	Best haplotype match	Top Hit	% Homol	Best match (dbs without species designation)
F02_MS	COI	GU074891.1	746/747 (99%)		Arthropoda-Ixodida-Ixodes scapularis	100.0	
F03_MS	COI	GU074891.1	729/747 (98%)		Arthropoda-Ixodida-Ixodes scapularis	98.25	
	16S	L43869.1	330/337 (98%)	isolate 5, strain Oklahoma			
F04_MS	COI	GU074891.1	745/747 (99%)		Arthropoda-Ixodida-Ixodes scapularis	100.0	
F05_MS	COI	GU074891.1	729/747 (98%)		Arthropoda-Ixodida-Ixodes scapularis	98.25	
F06_MS	COI	GU074891.1	703/746 (94%)		No match		Arthropoda-Ixodidae-Ixodes Canada (Quebec)
	16S	L43866.1	336/337 (99%)	isolate 3, strain Georgia 4			
		AF309011.1	336/337 (99%)	haplotype SSCP M			
F08_MS	COI	GU074891.1	703/746 (94%)		No match		Arthropoda-Ixodidae-Ixodes Canada (Quebec)
F09_MS	COI	GU074891.1	712/745 (96%)		Arthropoda-Ixodida	97.33	
F11_MS	COI	GU074891.1	698/747 (93%)		No match		Arthropoda-Ixodidae-Ixodes Canada (Quebec)
G02_MS	COI	GU074891.1	746/747 (99%)		Arthropoda-Ixodida-Ixodes scapularis	100.0	
	16S	FR854228.1	336/336 (100%)	Central Saskatchewan, Canada/**			
G03_MS	COI	GU074891.1	731/747 (98%)		Arthropoda-Ixodida-Ixodes scapularis	98.47	
	16S	FR799029.1/**	337/337 (100%)	Canada/**			
G04_MS	COI	GU074891.1	727/747 (97%)		Arthropoda-Ixodida-Ixodes scapularis	97.99	
	16S	L43869.1	331/337 (98%)	isolate 5, strain Oklahoma			
G05_MS	COI	GU074891.1	727/747 (97%)		Arthropoda-Ixodida-Ixodes scapularis	98.23	
G06_MS	COI	GU074891.1	728/747 (97%)		Arthropoda-Ixodida-Ixodes scapularis	98.48	
G07_MS	COI	GU074891.1	743/747 (99%)		Arthropoda-Ixodida-Ixodes scapularis	98.96	
	16S	FR799029.1/**	335/337 (99%)	Canada/**			
G08_MS	COI	GU074891.1	745/747 (99%)		Arthropoda-Ixodida-Ixodes scapularis	97.91	
G09_MS	COI	GU074891.1	736/747 (99%)		Arthropoda-Ixodida-Ixodes scapularis	98.67	
	16S	L43869.1	336/337 (99%)	isolate 5, strain Oklahoma			
G10_MS	COI	GU074891.1	741/747 (99%)		Arthropoda-Ixodida-Ixodes scapularis	100.0	
G11_MS	COI	GU074891.1	698/747 (93%)		No match		Arthropoda-Ixodidae-Ixodes Canada (Quebec)
	16S	AF309013.1	337/337 (100%)	SSCP O, North Carolina			
MSUB	16S	L43869.1	331/337 (98%)	isolate 5, strain Oklahoma			
MSUD	16S	L43855.1	336/337 (99%)	isolate 1, strain Georgia 3			

**Nearest match to sample from USA = U26605.1, Ixodes dammini strain IL94, Illinois.

### Phylogenetics

**Figure 2:**
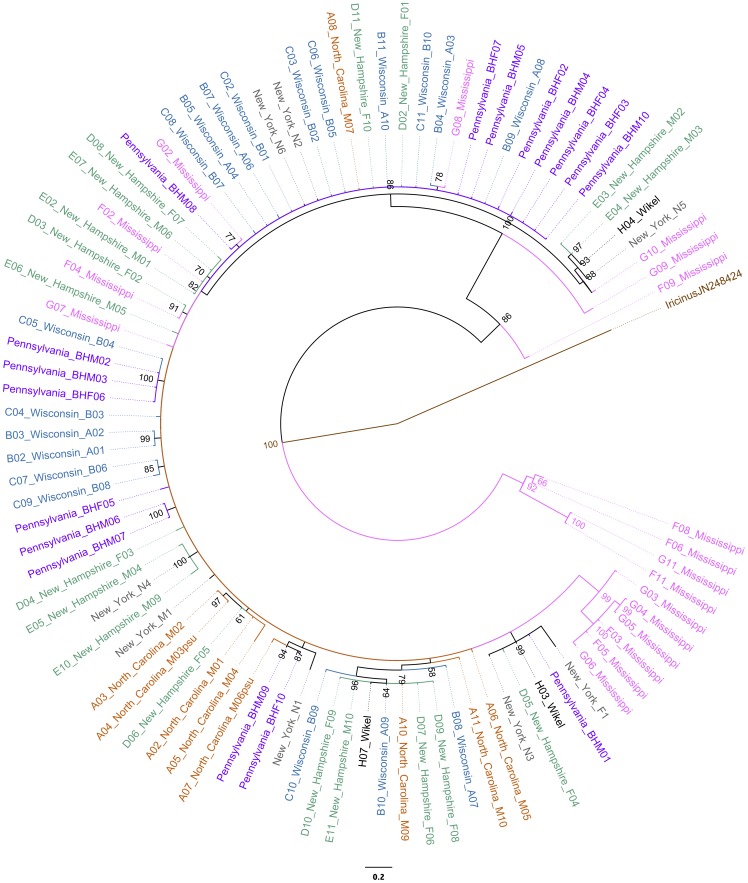
*I. scapularis* COI Bayesian phylogeny. The tree was rooted to *Ixodes ricinus* ( =  Brown, GenBank JN248424). Pink  =  Mississippi; blue  =  Wisconsin; orange  =  North Carolina; green  =  New Hampshire; purple  =  Pennsylvania; gray  =  New York; and black  =  Wikel Colony. Numbers at nodes represent posterior probability values and branch length corresponds to number of substitutions.

Phylogenetic analysis was conducted on each gene, and on concatenated sequences of the three nuclear genes. In the COI tree, many of the MS samples clustered into two clades that were highly divergent from the other sequences (3%–7%) (Table 3, Figure 2). This amount of divergence is significantly greater than the BOLD <1% or <3% divergence criterion for assigning species or genus level designation. We next confirmed that the sequence divergence was specific to our Mississippi samples by comparing all our samples to other COI sequences that were available on GenBank (Table S1, Figure S1).

**Figure 3:**
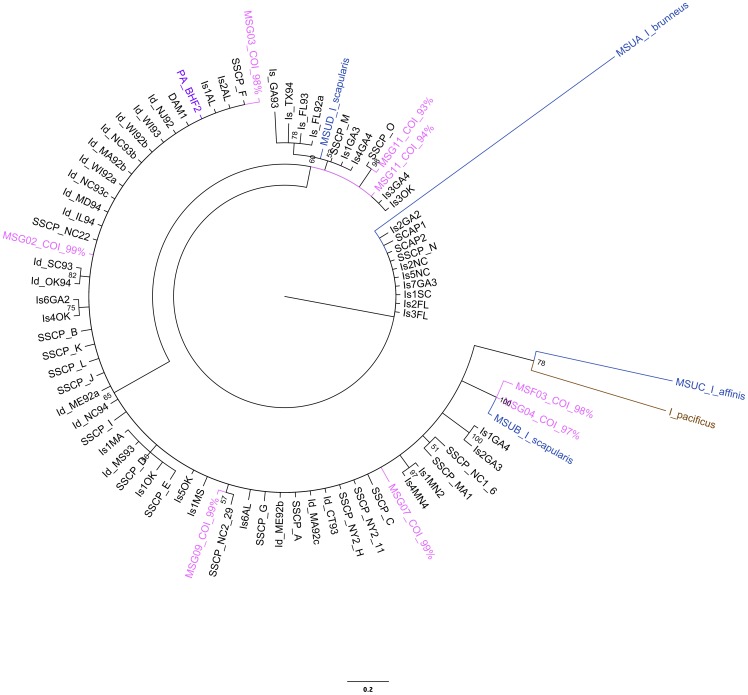
*I. scapularis* 16S Bayesian phylogeny with placement of MS unknowns. Tree includes all four MS unknowns and was left unrooted. Tree B was rooted to *I. pacificus* ( = brown) and included only sequences confirmed by blastn to be *Ixodes scapularis*. Pink = Mississippi samples; purple  =  Pennsylvania; red  =  specimens were blinded until after sequence confirmation. Numbers at nodes represent posterior probability values and branch length corresponds to number of substitutions.

**Figure 4:**
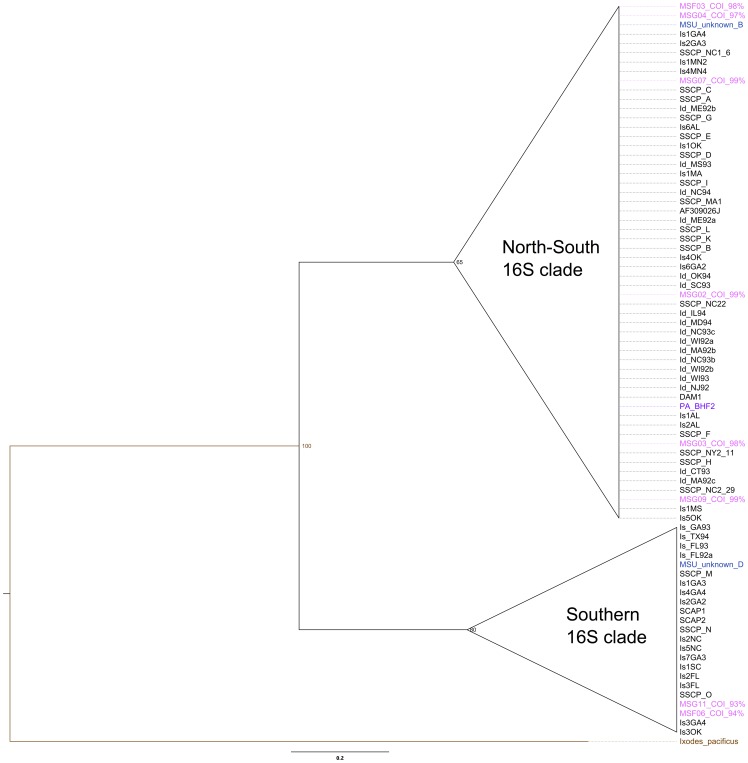
*I. scapularis* 16S Bayesian phylogeny compared to other *I. scapularis* sequences from Genbank. Tree is rooted to *I. pacificus* ( = brown) and included only sequences confirmed by blastn to be *Ixodes scapularis*. Pink = Mississippi samples; purple  =  Pennsylvania; red  =  specimens were blinded until after sequence confirmation. Numbers at nodes represent posterior probability values and branch length corresponds to number of substitutions.

To address whether we had misidentified some of the Mississippi samples, we cloned and sequenced a fragment of the 16S rRNA gene from a subset of divergent samples as well as known conspecifics from Mississippi. When we compared the sequences phylogenetically, it became evident that 1) there were several divergent haplotypes in Mississippi, 2) although COI clade separation was supported by 16S analysis, our divergent samples clustered with *I. scapularis* sequences from Genbank, and 3) though highly variable, both clades of *I. scapularis* 16S sequences were closer to each other than to the other congeneric sequences (Figures 3 and 4, Table S2).

**Figure 5:**
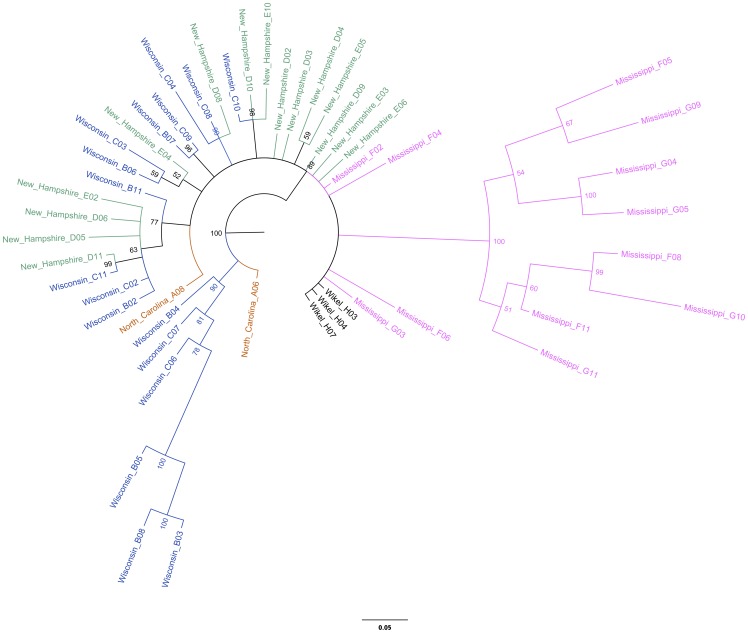
*I. scapularis* Bayesian phylogeny of concatenated nuclear sequences (see Figures S2, S3, and S4 for individual gene trees). Pink  =  Mississippi; blue  =  Wisconsin; orange  =  North Carolina; green  =  New Hampshire; purple  =  Pennsylvania; and black  =  Wikel Colony. Numbers at nodes represent posterior probability values and branch length corresponds to number of substitutions.

To clarify these results, we sequenced 3 additional nuclear genes (ixoderin B, lysozyme and serpin 2). We performed phylogenetic analysis on individual genes (Figures S2, S3, and S4) and also on concatenated nuclear sequences for all samples for which all three sequences were available (Figure 5). In the concatenated analysis, most of the Mississippi samples clustered together, apart from the other samples. This clustering was primarily due to the very strong support for the Mississippi clade in the lysozyme gene tree (Figure S3), which was not reflected in the other nuclear genes (Figures S2 and S4).

### Diversity measures and demographics

We identified 58 unique haplotypes in our COI data. The Shannon index for each population in our COI dataset ranged from 1.906 to 2.659 (Table 4). We analyzed the heterogeneity of alleles within the population using the haplotype genetic diversity index (h and uh) to estimate the probability that two haplotypes are different. The unbiased diversity probabilities ranged from 0.934 to 0.980, indicating high probabilities of haplotype diversity within populations (Table 4). In addition, we calculated the evenness to be between 0.7768 and 0.9299. Collectively these data indicate that, while the haplotype diversity was high, haplotypes were more or less evenly distributed within each population.

**Table 4:** Haplotype diversity of COI by population.

Population	N	Na	Ne	I	h	uh	Eh
**North Carolina**	9	8	7.364	2.043	0.864	0.972	0.9299
**Wisconsin**	19	15	10.94	2.580	0.909	0.959	0.8761
**New Hampshire**	18	13	10.80	2.476	0.907	0.961	0.8567
**Mississippi**	18	15	13.50	2.659	0.926	0.980	0.9201
**New York**	8	7	6.400	1.906	0.844	0.964	0.9167
**Pennsylvania**	17	10	8.257	2.201	0.879	0.934	0.7768
***Mean***	*14.83*	*11.33*	*9.543*	*2.311*	*0.888*	*0.962*	*0.8794*
***SE***	*2.023*	*1.430*	*1.087*	*0.125*	*0.013*	*0.006*	*0.0236*

*Diversity statistics were calculated using the Excel plugin Genalex v. 6.5.*

*N =  number of samples*;

*Na =  number of different alleles*;

*Ne = Number of effective alleles  =  1/Sum pi∧p2)*;

I = Shannon's Information Index  =  -1*Sum(pi*Ln(pi);

*h = heterozygosity/diversity  = 1-Sum pi∧2*;

uh  =  Unbiased heterozyosity/diversity  =  S/S-1))*h;

*Eh = Shannon's equitability = I/lnS*;

*pi is the frequency of the ith allele for the population & Sum pi∧2 is the sum of the squared population allele frequencies*.

*A high I indicates high diversity and evenness, while I = 0 indicates a single haplotype in a population. Equitability (Eh) ranges from 0-1, with 1 representing complete evenness in a population. Haploid genetic diversity index (h and uh) estimates the probability that two haplotypes will be different*.

**Figure 6:**
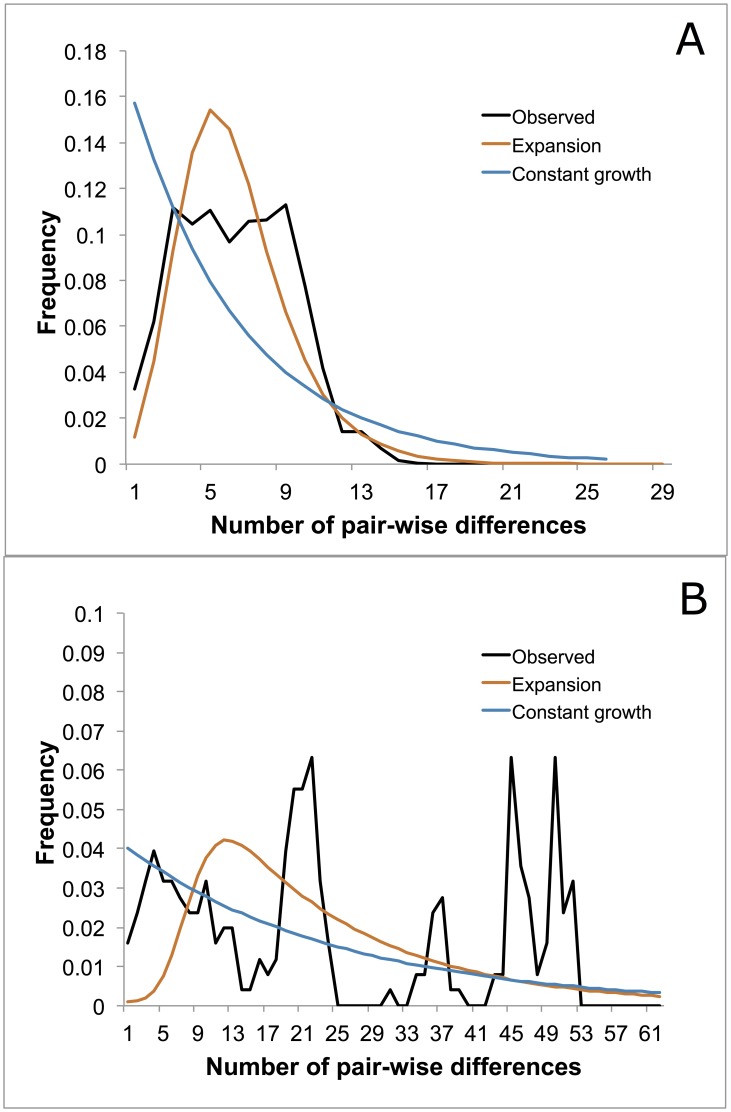
Mismatch distribution of observed frequencies of pairwise differences among *I. scapularis* COI sequences and expected frequencies under the neutral model of evolution given the null hypothesis of no population change or population expansion. A) Northern populations (WI, NY, NH, PA). B) Southern populations (MS, NC).

**Figure 7:**
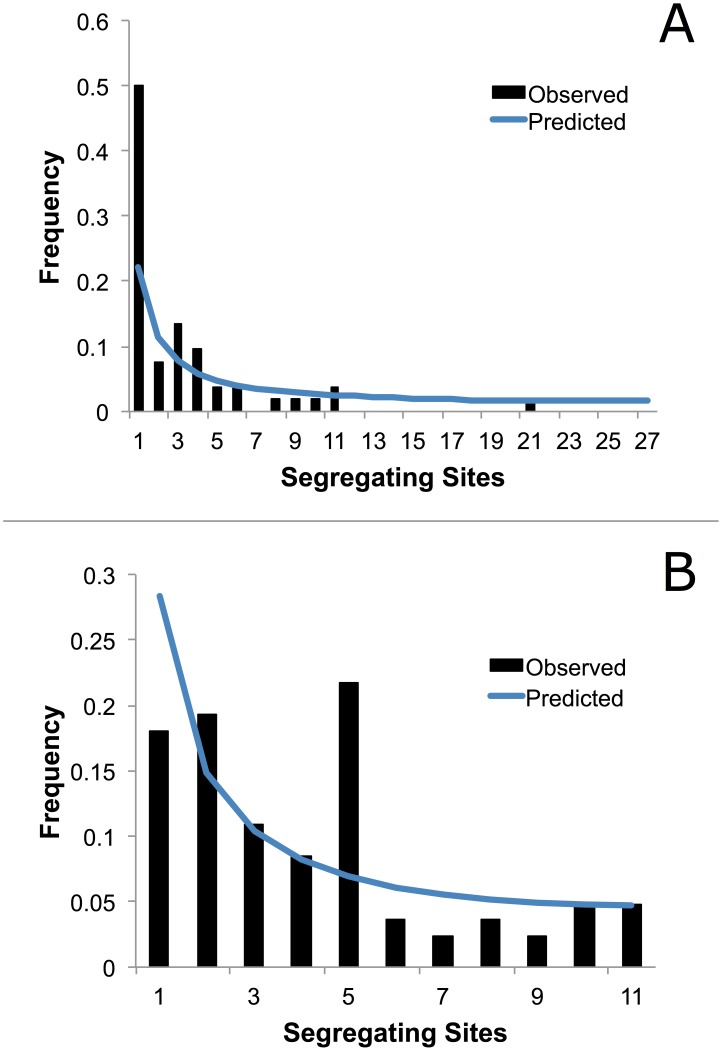
Site-frequency spectrum in *I. scapularis* COI sequences. Spectrum compares observed frequencies of segregating sites to expected distribution under the null hypothesis of no population change. A) Northern populations exhibit an excess of singleton mutations. B) Southern populations do not exhibit excess of singletons.

In the southern USA, COI sequences did not deviate statistically from neutrality based on Tajima's D test and Fu and Li's D* and F* tests (Table 5). However, in the Northern USA, Tajima's D and Fu and Li's D* and F* were significantly negative. Negative values for these statistics are often indicative of directional selection or population expansion. Population expansion was further supported by the mismatch distributions and site frequency spectra. The mismatch distribution for the Northern USA showed a peak that was consistent with population expansion (Figure 6A). The site frequency spectra exhibit an excess of singleton mutations, also consistent with population expansion (Figure 7A). For the southern USA, the mismatch distribution and site frequency spectra are not consistent with an expansion event (Figures 6B and 7B).

**Table 5:** Tests for neutrality for each gene.

						Tajima's		Fu and Li's			
Gene	Population	N	S	θ_π_	θ_S_	D	*P>0.05*	D*	*P>0.05*	F*	*P>0.05*
Ixoderin B	North Carolina	4	5	2.500	2.727	−0.7968	***0.0000***	−0.7968	***0.0000***	−0.7968	***0.0000***
	Wisconsin	17	22	6.471	6.507	−0.0226	*0.5447*	0.8968	*0.8606*	0.6712	*0.8917*
	New Hampshire	18	14	2.212	4.070	−1.7145	***0.0286***	−1.6295	*0.0798*	−1.7500	*0.0602*
	Mississippi	16	11	2.108	3.315	−1.3752	*0.0727*	−2.2241	***0.0152***	−2.0902	***0.0361***
	Northern USA (WI, NH)	35	30	4.501	7.285	−1.3443	*0.0706*	−0.1177	*0.4224*	−0.5800	*0.2708*
	Southern USA (NC, MS)	20	15	2.263	4.228	−1.7150	***0.0268***	−2.5753	***0.0105***	−2.4750	***0.0176***
Lysozyme	North Carolina	8	3	0.750	1.157	−1.4475	***0.0000***	−1.5653	***0.0000***	−1.5577	***0.0000***
	Wisconsin	20	5	0.589	1.409	−1.7800	***0.0163***	−2.0124	***0.0163***	−2.0622	***0.0163***
	New Hampshire	18	5	0.935	1.454	−1.1302	*0.1294*	−0.3590	*0.3099*	−0.6013	*0.2465*
	Mississippi	15	22	6.762	6.766	−0.0025	*0.5461*	0.0541	*0.4663*	0.0404	*0.4999*
	Northern USA (WI, NH)	38	6	0.744	1.428	−1.2929	*0.0906*	−0.4349	*0.2045*	−0.7637	*0.2190*
	Southern USA (NC, MS)	23	25	7.123	6.773	0.1933	*0.6394*	−0.6782	*0.2515*	−0.4436	*0.3107*
Serpin 2	North Carolina	6	4	1.733	1.752	−0.0572	*0.4403*	0.0713	*0.5352*	0.0441	*0.4403*
	Wisconsin	20	18	3.768	5.074	−0.9679	*0.1697*	−0.4847	*0.2969*	−0.6674	*0.2427*
	New Hampshire	17	19	3.941	5.620	−1.1771	*0.1117*	−1.3121	*0.0962*	−1.3450	*0.1055*
	Mississippi	17	23	6.206	6.803	−0.3514	*0.4020*	−0.1699	*0.3783*	−0.2340	*0.3882*
	Northern USA (WI, NH)	37	22	3.802	5.270	−0.9437	*0.1792*	0.7510	*0.8013*	0.2077	*0.6019*
	Southern USA (NC, MS)	23	26	5.352	7.045	−0.9045	*0.1890*	−0.3671	*0.3141*	−0.5712	*0.2655*
COI	North Carolina	9	20	5.444	7.359	−1.2793	*0.0993*	−1.2724	*0.1286*	−1.2994	*0.1175*
	Wisconsin	19	19	3.647	5.524	−1.3190	*0.0805*	−1.1090	*0.1213*	−1.2386	*0.1201*
	New Hampshire	18	30	6.562	8.722	−0.9947	*0.1606*	−1.0233	*0.1487*	−1.0766	*0.1422*
	Mississippi	18	74	24.60	21.51	0.5984	*0.7846*	1.1233	*0.9483*	1.0297	*0.9263*
	New York	8	33	8.893	12.72	−1.6023	***0.0274***	−1.7262	***0.0238***	−1.7219	***0.0279***
	Pennsylvania	17	20	4.000	5.916	−1.2817	*0.0886*	−0.6769	*0.2134*	−0.8960	*0.1842*
	Northern USA (WI, NH, NY, PA)	63	62	5.326	13.20	−2.0265	***0.0045***	−3.5358	***0.0086***	−3.3531	***0.0062***
	Southern USA (NC, MS)	27	83	20.02	21.53	−0.2724	*0.4489*	0.6115	*0.7613*	0.3535	*0.6767*

Tajima's D, and Fu and Li's D* and F* were calculated using the online Intrapop Neutrality Test calculator ( http://wwwabi.snv.jussieu.fr/achaz/neutralitytest.html, [*38*] ). N = # samples; S = # segregating sites, i.e. polymorphic nucleotides; θ_π_ =  #mutations (theta) per site from pi (nucleotide diversity); and θ_S_ =  theta per segregation site. Bold P-values indicate statistical significance based on 100,000 replicates with no recombination, and normalized by their standard deviations. Significant positive D/D*/F* indicates balancing selection or bottleneck; significant negative D/D*/F* indicates directional or expansion.

Nuclear genes indicated a more variable evolutionary history. Serpin 2 did not deviate from neutrality in any population assayed (Table 5). For lysozyme, Tajima's D and Fu and Li's D* and F* were significantly negative in WI and NC. Results for ixoderin B were not consistent between tests, although southern samples were significantly negative suggesting possible directional selection on this gene (Table 5).

### Population structure

Analysis of molecular variance (AMOVA) was conducted on each gene. Obtained sequences were highly variable (Table 6). For all genes, the majority of variation was explained by variation between individuals within populations (41.6%–89.5%). However, our analysis also showed that collection region (North or South) explained significant variation for most tested genes. For the mitochondrial COI gene, and for 2 nuclear genes (lysozyme and serpin 2), a significant proportion of molecular variation was explained by Northern vs. Southern location (COI: 16.8%, P = 0.03; lysozyme: 37.5%, P = 0.026; serpin 2: 6.9%, P = 0.05). Although not significant, the trend for ixoderin B was similar (14.6%, P = 0.059) (Table 6).

**Table 6:** Analysis of molecular variance (AMOVA).

Gene	Source of Variation	d.f.	F statistic	F stat Value	SS	Variance	% Explained	*P*
COI	Among regions	1	Fct	0.168	50.9	1.059	16.77	0.03
	Among populations within regions	7	Fsc	0.127	77.9	0.667	10.56	<0.0001
	Within populations	80	Fst	0.273	366.9	4.587	72.67	<0.0001
	Total	88			495.7	6.313	100	
Lysozyme	Among regions	1	Fct	0.374	34.9	1.013	37.45	0.026
	Among populations within regions	5	Fsc	0.335	30.4	0.567	20.96	<0.0001
	Within populations	54	Fst	0.584	60.7	1.125	41.59	<0.0001
	Total	60			126	2.705	100	
Ixoderin B	Among regions	1	Fct	0.146	12.37	0.302	14.59	0.059
	Among populations within regions	5	Fsc	0.244	23.46	0.431	20.82	<0.0001
	Within populations	48	Fst	0.354	64.1	1.335	64.59	<0.0001
	Total	54			99.93	2.068	100	
Serpin 2	Among regions	1	Fct	0.069	7.37	0.161	6.86	0.05
	Among populations within regions	5	Fsc	0.039	14.1	0.085	3.64	0.076
	Within populations	53	Fst	0.105	110.95	2.093	89.5	<0.0001
	Total	59			132.42	2.339	100	

*Bold font in P-value column signifies statistical significance. Fct tests the genetic variability found among Northern versus Southern regions, Fsc tests genetic variability found among populations within Northern or Southern regions, and Fst tests genetic variability found among populations*.

### Population genetic distance

Mantel tests were conducted on COI, and the three nuclear genes, but no significant isolation by distance was detected. Due to the limited number of populations sampled, it is likely there is not enough power to detect a statistically significant relationship.

## Discussion

It has been long established that there are differences between northern and southern populations of *I. scapularis* in the United States [43]. At one time, they were considered by some groups to be two distinct species, based on biology and collection location (*I. scapularis* in the south and *I. dammini* in the north) [20]. Northern ticks have a 2-year life cycle and feed primarily on mammalian hosts, while the Southern ticks complete their lifecycles in a year or less depending on environmental conditions and feed primarily on lizards [44]. In addition, nymphs of southern populations of *I. scapularis* rarely bite humans and seem to quest in or beneath leaf litter instead of several inches above ground like those in northern areas [45] [46] [47] [48] [49] [50]. In 1993, Oliver et al designated *I. dammini* as a junior synonym of *I. scapularis* based on reciprocal crosses, assortative mating experiments, and morphometric criteria [18]. Subsequently, molecular studies confirmed that, while some data support distinct northern and southern lineages (using both mitochondrial 16S and 12S rRNA genes, and nuclear ITS1 and ITS2 regions of the ribosomal genes), there was insufficient evidence for isolation of the two morphotypes [19] [9] [14] [3] [12].

Similar to other studies, our mitochondrial and nuclear gene data support the division of *Ixodes scapularis* into several distinct lineages. We identified one clade that occurred only in southern population samples, and another clade that occurs throughout the northern and southern collection region. Our results suggest that northern and southern populations have significantly different demographic histories. Data from previous studies using 16S sequences have been used to infer that *I. scapularis* possibly originated in the southern USA, followed by migration of a small founder population into the north when the Laurentide ice sheet (Pleistocene era) receded [51] [12]. Our COI sequence data support this hypothesis. We detected statistical signatures of population bottleneck and expansion in northern populations, but not in southern populations (Figures 6 and 7, Table 5). Population bottlenecks and resultant expansions coincident with Pleistocene glaciation events have been detected in other vector taxa [52] [53] [43]. We speculate that demographic history may explain genetic differences between northern and southern *I. scapularis* populations.

Our results spotlight a potential hazard of relying solely on COI for species identification (“barcoding”) and population genetics. The COI gene has been shown to be a powerful tool for identification of species or cryptic species [54] [55]. There have been criticisms about dependence on a single gene for identification, particularly mitochondrial genes, in part because the evolution of mitochondrial genes may be influenced by endosymbionts [27] [26]. Because of the extent of the sequence divergence in our Mississippi samples, we might have concluded (on COI sequences alone) that we had sequenced 2 different species, or even 2 different genera. However, further confirmation with additional genes revealed that, while these samples were very divergent at the COI locus, they were, in fact, *I. scapularis*.

One explanation for the deep COI variation we observed in MS populations might be due to the presence of cryptic diversity within *I. scapularis*. A cryptic subspecies would be one that is morphologically similar to another, not reproductively isolated, but may have some genetic difference(s) that can phylogenetically separate the two. In *I. uriae* (seabird tick), Kempf et al 2009 compared microsatellite data (ecological timeframe) to COIII sequence data (evolutionary timeframe) to determine the timeframe of cryptic race divergence within *Ixodes uriae* and concluded that there was evidence of a cryptic race complex correlated with seabird hosts [56]. This geographical/host-associated variability was not previously detected with more slowly evolving genes such as cytB and nuclear ITS-2, suggesting that the evolution of these variants was a recent occurrence, and possibly may have arisen multiple times [56]. Whether our data indicate a similar cryptic race complex within *I. scapularis* will require more scrutiny of a much larger dataset (i.e. more individuals from many more populations from southern locations in the United States).

Another possible hypothesis to explain the COI divergence of southern populations of *I. scapularis* in the United States may be that the cytoplasmically-linked *Rickettsia* might play a role. Due to maternal inheritance, mitochondrial lineages can hitchhike with cytoplasmically inherited endosymbionts, leading to structuring of mitochondrial sequences that do not correlate with population differentiation [57] [26]. Although the frequency of the *I. scapularis Rickettsia* endosymbiont has been reported in ranges from 40–100% [23] [24], our own samples were 100% infected. However, in this study we did not sequence genes from the *Rickettsia* endosymbiont, so we do not have genetic data on the endosymbiont populations to support or refute the involvement of the *Rickettsia* in host COI divergence. It is unclear what the effect of the symbiont is on *I. scapularis* biology and evolution. We are currently conducting analyses of the endosymbiont population genetic structure. These data will be overlaid on vector population structure to possibly address whether or not this (or other symbionts) are affecting tick population structure.

Other hypotheses that might possibly explain the COI variation include nuclear genes of mitochondrial origin (“numts”), paternal mitochondrial leakage or mitochondrial heteroplasmy. “Numts” are instances of mitochondrial DNA that have been inserted into the nuclear genome and are therefore under different selection pressures [58]. We did not find that our COI sequences matched anything outside of the mitochondrial DNA when compared to the *I. scapularis* genome. Paternal mitochondrial leakage has been described in several systems including *Drosophila simulans*, in which 0.66% of 4092 offspring contained paternal mitochondrial COI [59], but has not been described in ticks. Relatively high levels of mitochondrial heteroplasmy have been observed in the tick *Amblyomma cajennense* [60], but the phenomenon has yet to be described in *Ixodes*.

Further in-depth population studies into more populations dispersed throughout the eastern seaboard and states east of the Mississippi would appear to be in order. In addition to the markers we have described here, there are others that could, collectively provide a more robust data set. With the advent of next generation sequencing and overall cost of sequencing becoming more affordable, it will be possible to conduct multi-gene sequencing studies on a population-wide scale.

## Supporting Information

Figure S1 **Comparison of** ***I. scapularis*** **Bayesian phylogeny of the COI sequences from this study, Genbank, and the** ***I. scapularis*** **genome.** The fragment was trimmed to an overlapping region of 338 bp prior to analysis. Numbers at nodes represent posterior probability values and branch length corresponds to number of substitutions.
(PDF)

Figure S2 ***I. scapularis*** **Bayesian phylogeny of ixoderin B gene sequences.** Numbers at nodes represent posterior probability values and branch length corresponds to number of substitutions.
(PDF)

Figure S3 ***I. scapularis*** **Bayesian phylogeny of lysozyme gene sequences.** Numbers at nodes represent posterior probability values and branch length corresponds to number of substitutions.
(PDF)

Figure S4 ***I. scapularis*** **Bayesian phylogeny of serpin 2 gene sequences.** Numbers at nodes represent posterior probability values and branch length corresponds to number of substitutions.
(PDF)

Table S1 **COI sequences used for comparisons.**
(XLSX)

Table S2 **16S sequences used for comparisons.**
(XLSX)
