# Supplementary figures and images for: Population and Demographic Structure of Ixodes scapularis Say in the Eastern United States
Source: PLoS One. 2014 Jul 15;9(7):e101389. doi: 10.1371/journal.pone.0101389 (PMC4099084; doi:10.1371/journal.pone.0101389)

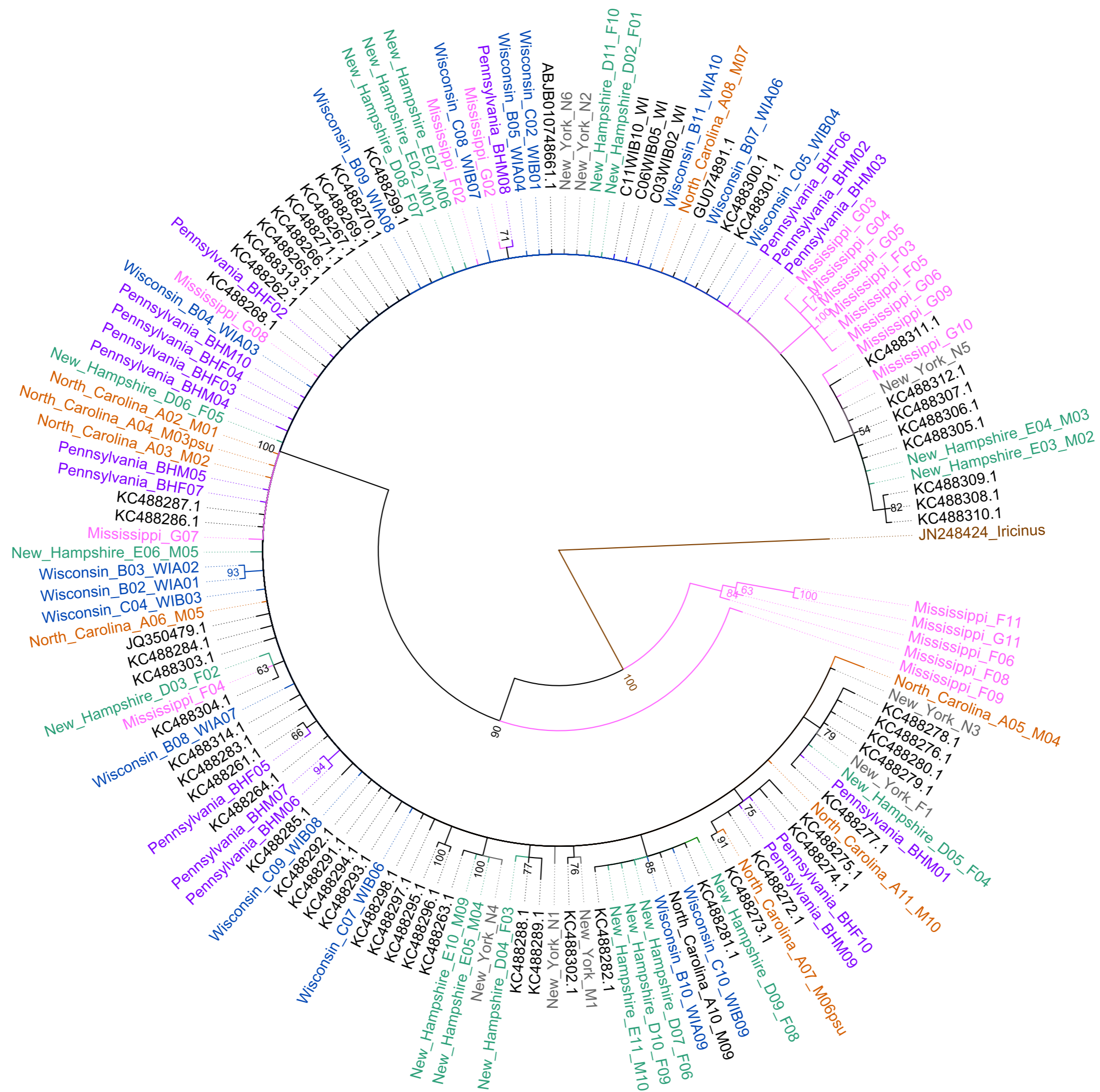

Supplement: Figure S1 — Comparison of I. scapularis Bayesian phylogeny of the COI sequences from this study, Genbank, and the I. scapularis genome. The fragment was trimmed to an overlapping region of 338 bp prior to analysis. Numbers at nodes represent posterior probability values and branch length corresponds to number of substitutions. (PDF) [file pone.0101389.s001.pdf]

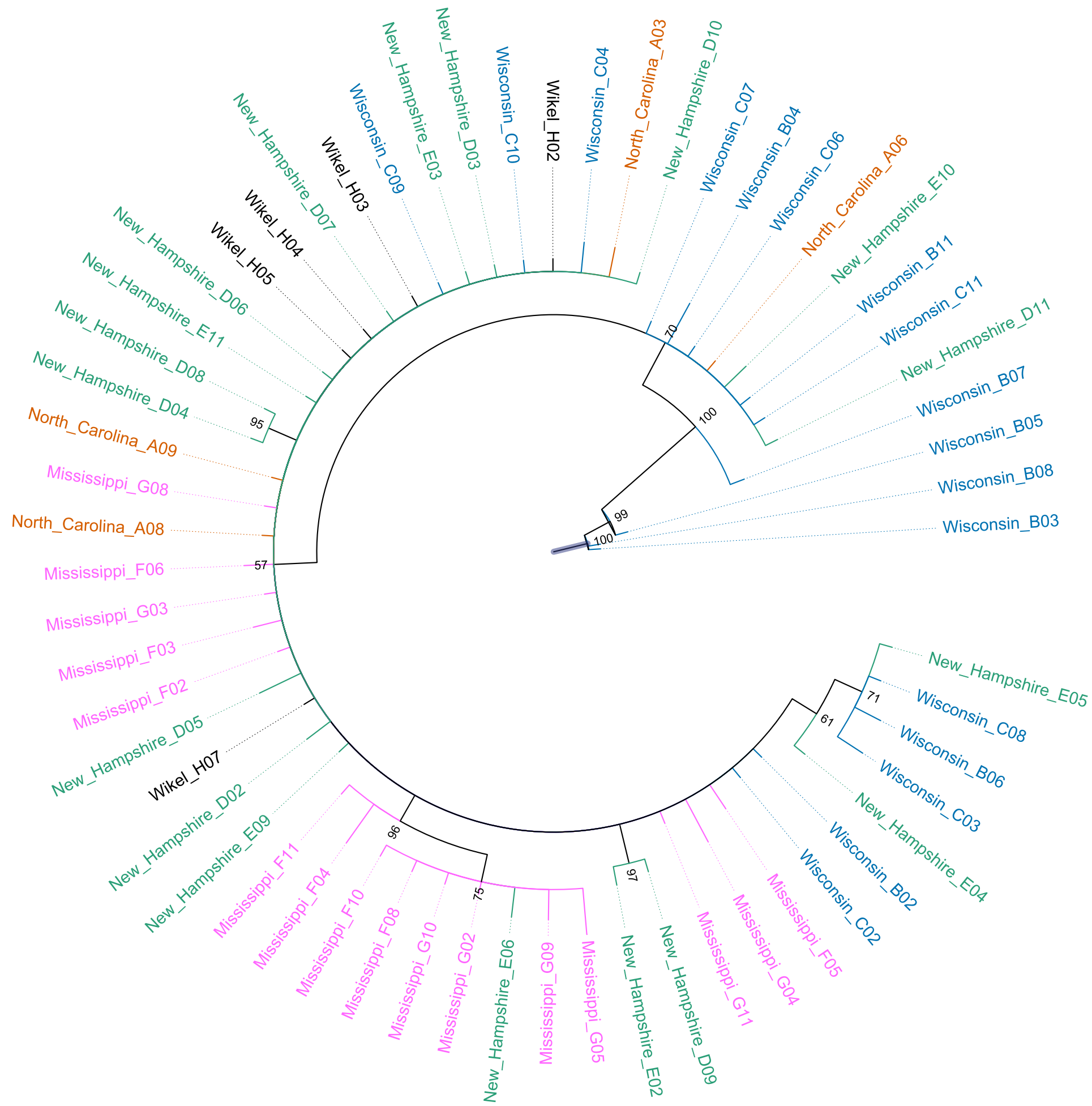

0.0050

Supplement: Figure S2 — I. scapularis Bayesian phylogeny of ixoderin B gene sequences. Numbers at nodes represent posterior probability values and branch length corresponds to number of substitutions. (PDF) [file pone.0101389.s002.pdf]

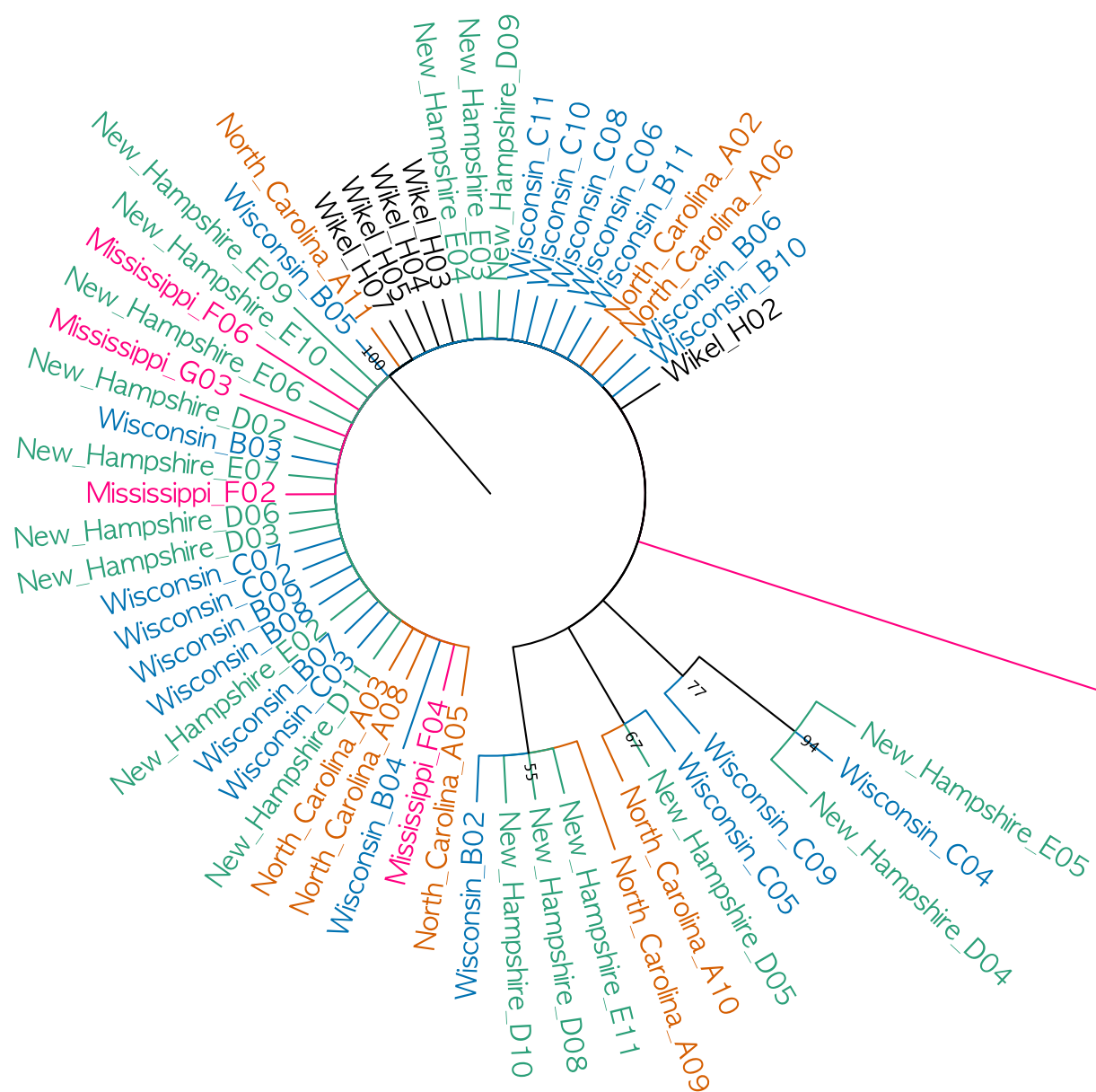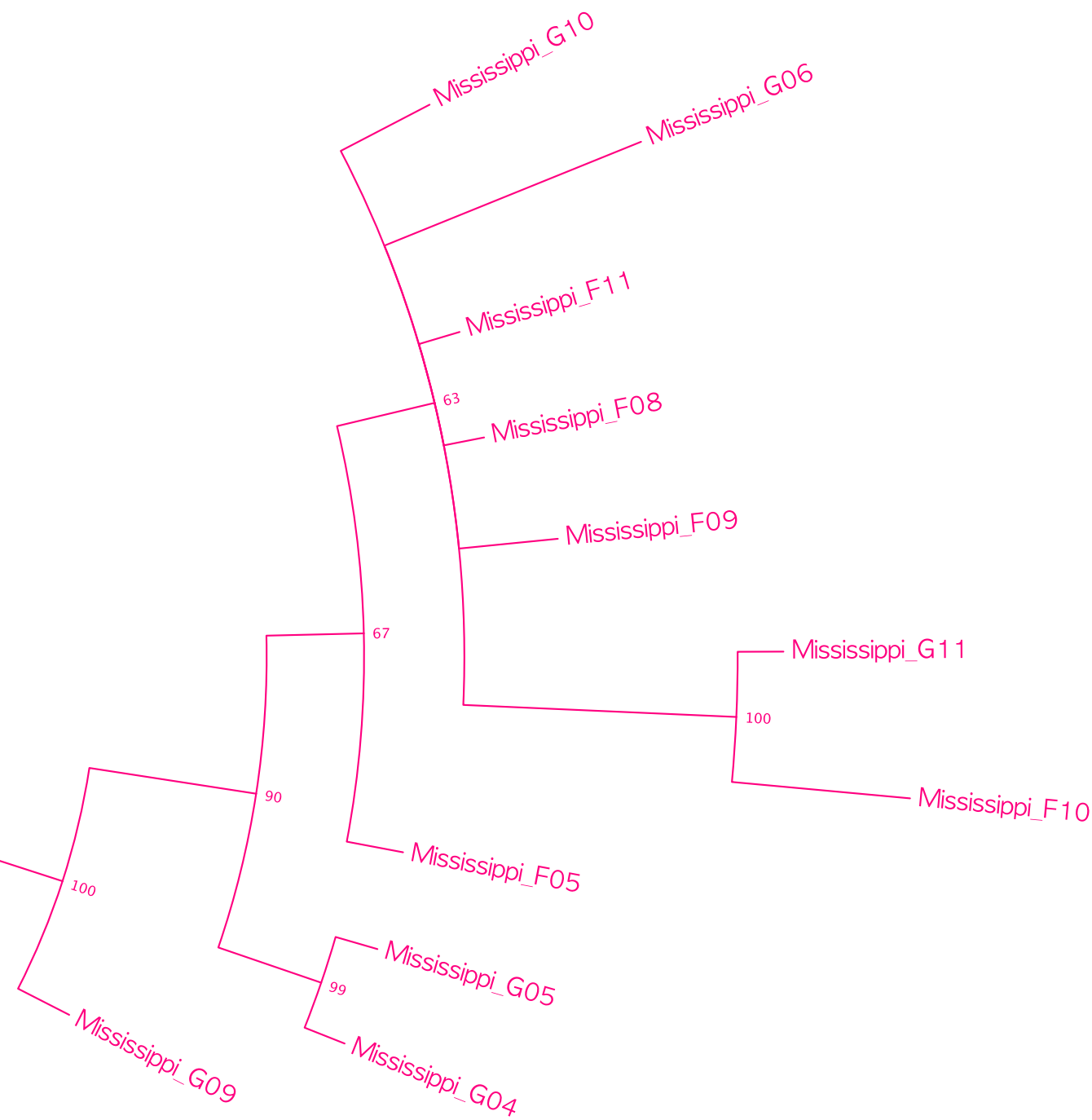

0.2

Supplement: Figure S3 — I. scapularis Bayesian phylogeny of lysozyme gene sequences. Numbers at nodes represent posterior probability values and branch length corresponds to number of substitutions. (PDF) [file pone.0101389.s003.pdf]

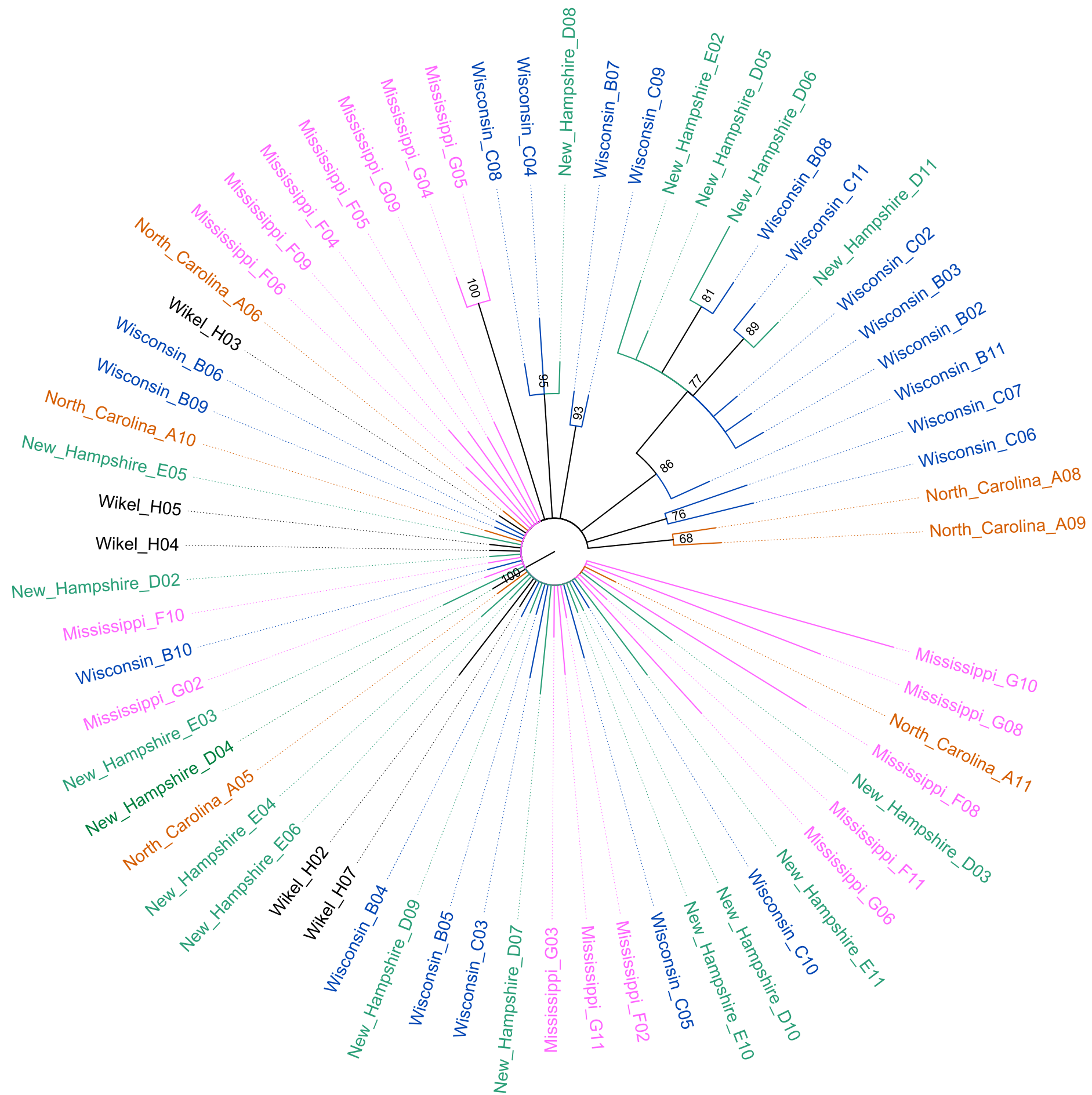

0.0030

Supplement: Figure S4 — I. scapularis Bayesian phylogeny of serpin 2 gene sequences. Numbers at nodes represent posterior probability values and branch length corresponds to number of substitutions. (PDF) [file pone.0101389.s004.pdf]
